# Big Data and Real-World Data based Cost-Effectiveness Studies and Decision-making Models: A Systematic Review and Analysis

**DOI:** 10.3389/fphar.2021.700012

**Published:** 2021-10-19

**Authors:** Z. Kevin Lu, Xiaomo Xiong, Taiying Lee, Jun Wu, Jing Yuan, Bin Jiang

**Affiliations:** ^1^ Department of Clinical Pharmacy and Outcomes Sciences, University of South Carolina, Columbia, SC, United States; ^2^ Department of Pharmaceutical and Administrative Sciences, Presbyterian College School of Pharmacy, Clinton, SC, United States; ^3^ Department of Clinical Pharmacy, School of Pharmacy, Fudan University, Shanghai, China; ^4^ Department of Administrative and Clinical Pharmacy, School of Pharmaceutical Sciences, Health Science Center, Peking University, Beijing, China

**Keywords:** big data, real-world data, cost-effectiveness analysis, pharmacoeconomics, systematic review

## Abstract

**Background:** Big data and real-world data (RWD) have been increasingly used to measure the effectiveness and costs in cost-effectiveness analysis (CEA). However, the characteristics and methodologies of CEA based on big data and RWD remain unknown. The objectives of this study were to review the characteristics and methodologies of the CEA studies based on big data and RWD and to compare the characteristics and methodologies between the CEA studies with or without decision-analytic models. **Methods:** The literature search was conducted in Medline (Pubmed), Embase, Web of Science, and Cochrane Library (as of June 2020). Full CEA studies with an incremental analysis that used big data and RWD for both effectiveness and costs written in English were included. There were no restrictions regarding publication date. **Results:** 70 studies on CEA using RWD (37 with decision-analytic models and 33 without) were included. The majority of the studies were published between 2011 and 2020, and the number of CEA based on RWD has been increasing over the years. Few CEA studies used big data. Pharmacological interventions were the most frequently studied intervention, and they were more frequently evaluated by the studies without decision-analytic models, while those with the model focused on treatment regimen. Compared to CEA studies using decision-analytic models, both effectiveness and costs of those using the model were more likely to be obtained from literature review. All the studies using decision-analytic models included sensitivity analyses, while four studies no using the model neither used sensitivity analysis nor controlled for confounders. **Conclusion:** The review shows that RWD has been increasingly applied in conducting the cost-effectiveness analysis. However, few CEA studies are based on big data. In future CEA studies using big data and RWD, it is encouraged to control confounders and to discount in long-term research when decision-analytic models are not used.

## Background

With the development of health care technologies, a large number of innovative medications and health-related interventions have been approved and available on the market (Claxton et al., 2011; Hughes-Wilson et al., 2012). While these new therapies deliver better health outcomes, they often come with additional economic burdens (Hughes-Wilson et al., 2012). The cost-effectiveness analysis (CEA) is one of the economic evaluation techniques comparing both outcomes and costs between two or more interventions, which could help decision-makers to decide the most appropriate intervention and help payers to estimate the economic burden (Eichler et al., 2004; Drummond and McGuire, 2005; Bowrin et al., 2019). When the effectiveness is measured by a utility, it is called cost-utility analysis (CUA) (Drummond and McGuire, 2005). CEA has been increasingly used by health technology assessment (HTA) agencies in many countries for the decision-making of health-related interventions, including but not limited to market access, pricing, and formulary (Yang et al., 2008; Clement et al., 2009; Ciani and Jommi, 2014; Dakin et al., 2015; Jönsson, 2015).

CEA can be directly performed based on randomized controlled trials (RCTs) or pragmatic studies, or it can be indirectly conducted using decision-analytic models with mixed data derived from RCTs and the real-world settings (Drummond and McGuire, 2005; Briggs et al., 2006). Decision-analytic models, such as the decision tree and the Markov model, are a systematic decision-making approach widely using in the economic evaluation of healthcare interventions to compare decisions under uncertainty (Drummond and McGuire, 2005; Briggs et al., 2006). Real-world data (RWD) provided by observational studies other sources, including medical claims data and electronic health records (EHRs) have been used more and more in CEA studies (Drummond, 1996; Terkola et al., 2017; Bowrin et al., 2019). The International Society for Pharmacoeconomics and Outcomes Research (ISPOR) Real-World Data Task Force published a report supporting the use of RWD for coverage and payment decisions in 2007, which defined RWD as the data used not collected in conventional RCTs (Garrison et al., 2007). Specifically, six sources of RWD were defined by the ISPOR, including supplements to traditional RCTs, large simple trials, registries, administrative data, health surveys, and EHRs and medical chart reviews. In 2017, ISPOR and International Society for Pharmacoepidemiology (ISPE) Joint Task Force published an article about the practice for real-world studies of comparative effectiveness (Berger et al., 2017). Compared to the RCTs considered as the “golden standard” in evaluating efficacies, RWD from observational studies or other real-world settings features a larger sample size (Silverman, 2009; Makady et al., 2018). Additionally, real-world settings can offer long-term scrutinization of effectiveness, which is reliable and ensures less uncertainty in a lifetime decision-analytic model compared to the RCTs commonly designed with a relatively short time horizon (Makady et al., 2018; Bowrin et al., 2019).

With the evolvement of technology, big data have been used more and more often in health care settings. Big data are a special kind of real-world data, which are characterized by high volume, high velocity, high variety, high value, and high veracity (5Vs) (Mehta and Pandit, 2018). Big data combine data from a variety of sources, including insurance claims, electronic medical records, patient-reported data, social media, etc. The combined data can be analyzed to predict the diagnosis and medication administration patterns using artificial intelligence models such as machine learning to compare health-related interventions (Wordsworth et al., 2018). However, because many big data are unstructured, certain challenges in the data collection, management, cleaning, and analysis need to be addressed before big data can be widely used in CEA studies (Wordsworth et al., 2018).

Limited studies have systematically reviewed the characteristics, methodologies, and quality of CEA studies based on big data and RWD. A study in 2019 reviewed the limitations in using RWD for CEA studies (Mehta and Pandit, 2018). However, this review does not include specific CEA studies using RWD, but overview literature (Mehta and Pandit, 2018). In addition, no studies have examined the differences between CEA studies based on big data and RWD with or without decision-analytic models. To fill the gap in the literature, the objectives of this study were to review the characteristics and methodologies of the cost-effectiveness analysis based on big data and real-world data and to compare the characteristics and methodologies between the cost-effectiveness analyses with or without decision-analytic models.

## Methods

### Search Strategy and Sources

A comprehensive literature search was implemented to identify CEA studies using big data and RWD. The literature search was conducted within the scope of four databases (as of June 2020) including Medline (Pubmed), Embase, Web of Science, and Cochrane Library. In addition, manual searches on the reference lists of included studies as well as related systematic reviews were performed to ensure the retrieval completeness. Search terms used in this study include cost-effectiveness analysis, cost-utility analysis, economic evaluation, pharmacoeconomics, big data, real-world study, real-world evidence, real-world data, RWD, RWE, RWS, electronic health records, EHRs, claims, and registry. Details are shown in Supplemental Material S1.

### Eligibility Criteria

Full CEA studies with an incremental analysis that compared both incremental effectiveness and incremental cost between two or more interventions that used big data and RWD for both effectiveness and costs written in English were included. The definition of RWD in this review was based on the report published by ISPOR, where RWD was defined as data not derived from RCTs but rather come from pragmatic trials, registries, administrative data, health surveys, electronic records, or paper medical charts (Garrison et al., 2007). Big data were identified if two or more RWD were combined in a single parameter, or if any artificial intelligent methods, such as machine learning and deep learning methods, were used to process the data (Mehta and Pandit, 2018; Wordsworth et al., 2018). Furthermore, there were no restrictions regarding the publication date. Cost‐minimization analysis, cost-of-illness, cost-benefit analysis, reviews, meta‐analysis, comments, letters, protocols, posters or presentations at conferences or workshops, literature unavailable, and studies that are not health-related were excluded.

### Study Selection

According to the patient/population, intervention, comparison and outcomes (PICOS) principle, patients were any patients, data for intervention and control groups were from the real world, and outcomes were ICER (Amir-Behghadami and Janati, 2020). Two rounds of screening were carried out independently by two reviewers after removing duplicates. In case a disagreement was expressed, a senior reviewer made the final decision. In the first round, titles and abstracts were screened for eligibility. Studies were included if 1) baseline population is based on real-world studies; 2) big data and RWD are used for both effectiveness and costs; 3) transition probabilities in the decision-analytic models are not obtained from RCTs. Then, the full-text review was performed for verification of potentially eligible studies according to the eligibility criteria. The entire selection process, including study identification, eligibility screening, and selection of full-text articles, followed the preferred reporting items for systematic reviews and meta-analyses (PRISMA) statement (Moher et al., 2009).

### Information Extraction and Data Synthesis

**FIGURE 1:**
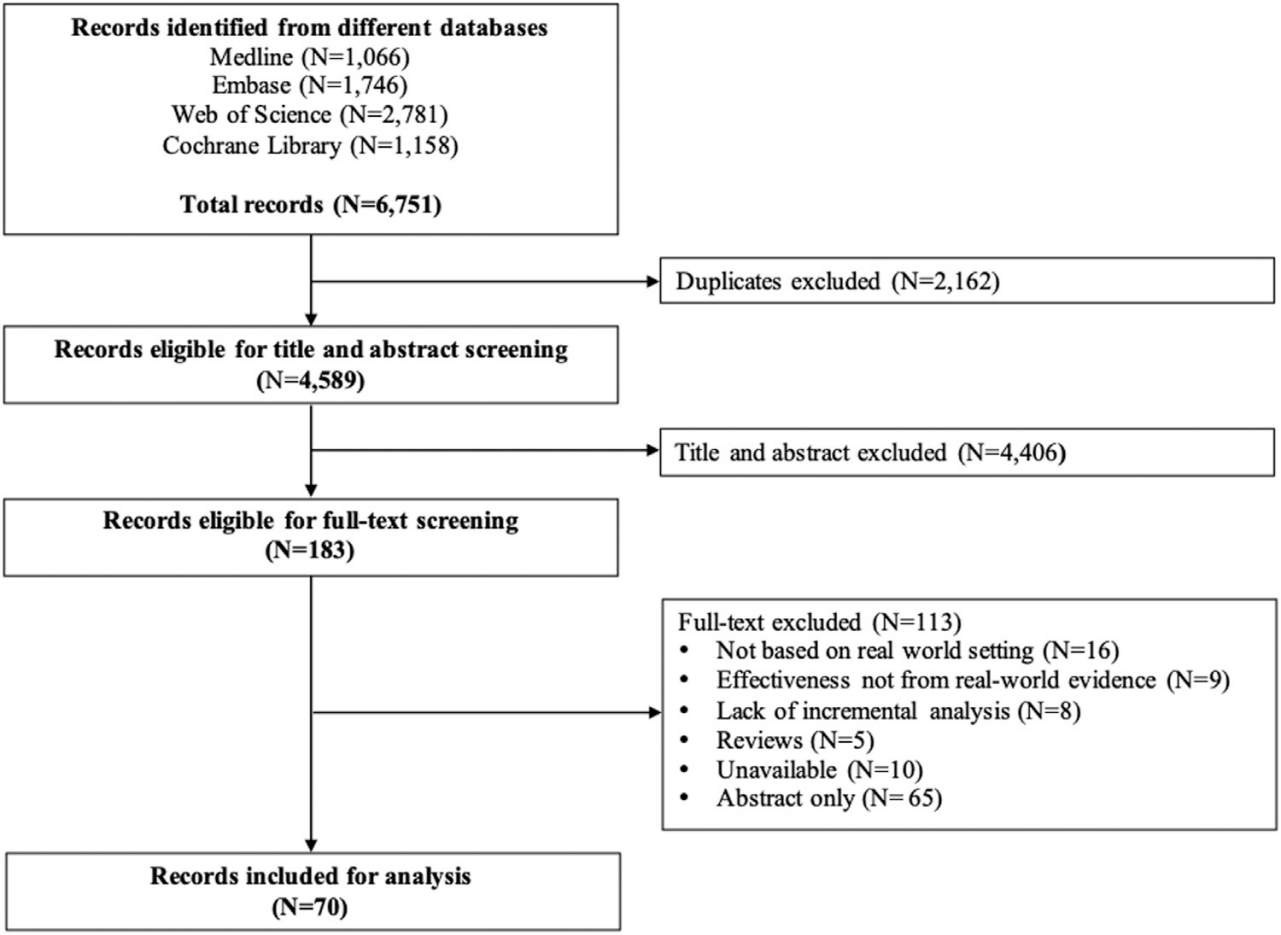
Flowchart of publication selection.

The extracted information included the study characteristics: title, name of the first author, published year, study regions, affiliations of the first author, funding sources, diseases, interventions, and sample size. We also collected information on the study methodologies: study design, data type (RWD or big data), time horizon, methods of controlling confounders, the primary outcome, indirect costs (including the cost of absence from paid work, reduced productivity at paid work, and unpaid production), sources of effectiveness, sources of costs, report of missing data, methods of handling missing data, threshold consideration, sensitivity analysis, and discount rate (Liljas, 1998; McNamee, 2005). The process of study selection, along with the included and excluded number of studies, was presented in a PRISMA flowchart (Figure 1). The descriptive characteristics and methodologies were summarized and compared between the cost-effectiveness analyses with or without decision-analytic models.

### Quality Assessment

We assessed the quality of the included studies using the Quality of Health Economic Studies (QHES) instrument (Ofman et al., 2003; Di Marco et al., 2018; McQueen et al., 2018). The QHES instrument consists of 16 items with scores ranging from 1 to 9, and the total score of the instrument is 100. During the assessment process, if the included study satisfied the criterion of an item, the study received an item-specific score, otherwise it received a score of zero. The quality assessment was conducted by two reviewers independently, and any controversies were resolved by discussion with a third investigator to reach a consensus. The QHES has a score-based grading system. The QHES has a score-based grading system. The scores are grouped into four groups: extremely poor quality (0–24), poor quality (25–49), fair quality (50–74), and high quality (75–100) (Ofman et al., 2003). Details of the QHES instrument are shown in Supplemental Material S2.

## Results

### Overview

**FIGURE 2:**
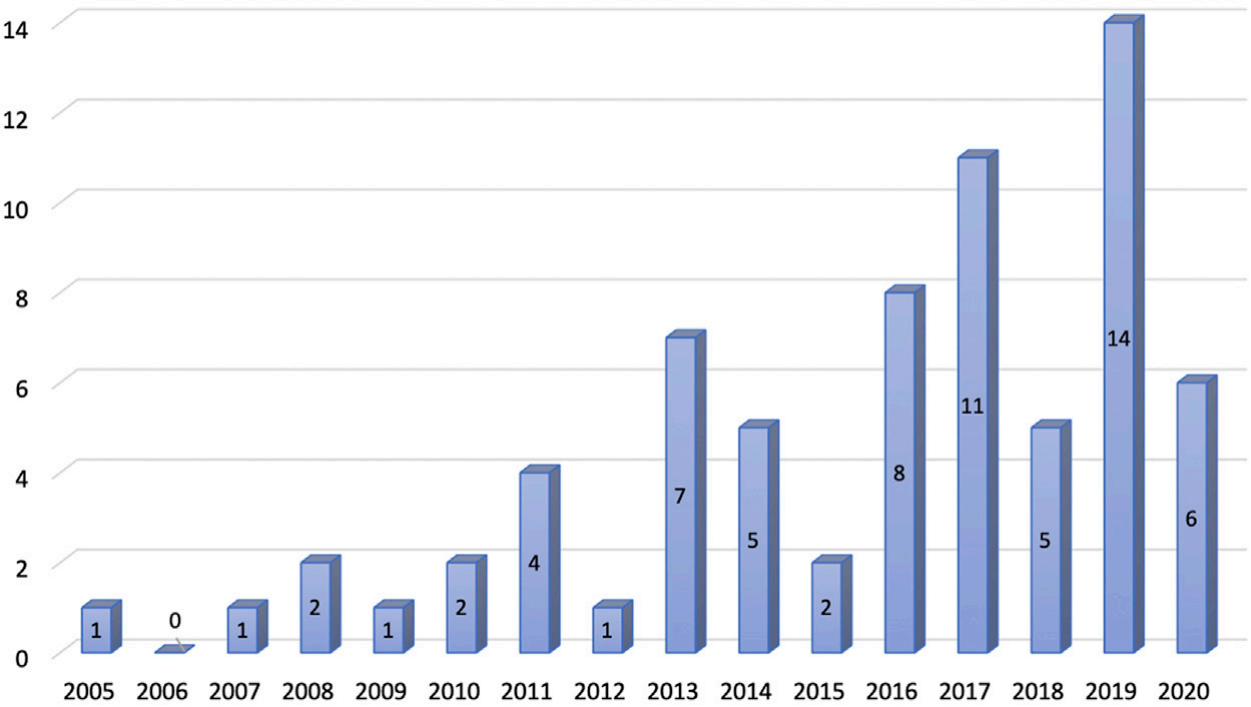
Trends in the publications of real-world based cost-effectiveness analysis.

The systematic literature search identified 6,751 studies after applying search strategies from different databases combined. After removing duplicates, 4,589 studies were eligible for the title and abstract screening. Upon screening of the titles and abstracts, 4,406 studies were excluded. The full-text screening was conducted on 183 eligible studies. A total of 113 studies were excluded from the full-text review, and a total of 70 studies were finally included for review (Figure 1). The number of publications on CEA studies based on big data and RWD increased over the years, and the majority of the studies were published between 2011 and 2020 (Figure 2).

### Charcteristics of Included Studies

Among 70 studies included, 37 (52.9%) were based on decision-analytic models, and 33 (47.1%) were not. The study regions of most studies were in Europe (42.9%). For the research design, the number of studies using CEA (55.7%) was slightly more than that of those using CUA (44.3%). Nearly 70% of the studies had a sample size higher than 100, and around a quarter of the stuides did not report the sample size. The most frequently used study perspective was the health care system (45.7%), followed by society (22.9%) and patients (11.4%). Most of the authors were from government or academic institutions (67.1%), and most of the funding came from the industry (48.6%). Neoplasms (25.7%) and circulation diseases (24.3%) were the most frequently studied diseases. The most frequently evaluated intervention was pharmacological treatment (54.3%). (Table 1).

**TABLE 1:** Characteristics of included studies.

Characteristics	Total *N* = 70		Analytic decision model *N* = 37		Non-analytic decision model *N* = 33	
	*N*	%	*N*	%	*N*	%
Year						
2000–2010	7	10.0	4	10.8	3	9.1
2011–2015	19	27.1	11	29.7	8	24.2
2016–2020	44	62.9	22	59.5	22	66.7
Study regions						
Africa	2	2.9	0	0.0	2	6.1
Asia	18	25.7	11	29.7	7	21.2
Europe	30	42.9	18	48.6	12	36.4
Oceania	2	2.9	2	5.4	0	0.0
North America	18	25.7	6	16.2	12	36.4
Study types						
CEA	31	44.3	5	13.5	26	78.8
CUA	39	55.7	32	86.5	7	21.2
Sample size						
0–100	4	5.7	0	0.0	4	12.1
101–500	18	25.7	10	27.0	8	24.2
501–1,000	9	12.9	5	13.5	4	12.1
1,001–10,000	13	18.6	4	10.8	9	27.3
≥10,001	8	11.4	2	5.4	6	18.2
NA	18	25.7	16	43.2	2	6.1
Cost perspectives						
Patients	8	11.4	3	8.1	5	15.2
Society	16	22.9	11	29.7	5	15.2
Health care system	32	45.7	17	45.9	15	45.5
Third-party payer	4	5.7	2	5.4	2	6.1
Others	3	4.3	2	5.4	1	3.0
NA	7	10.0	2	5.4	5	15.2
Affiliations of the first author						
Government/academia	47	67.1	26	70.3	21	63.6
Hospital	12	17.1	3	8.1	9	27.3
Industry	2	2.9	1	2.7	1	3.0
Consulting firms	9	12.9	7	18.9	2	6.1
Funding sources						
Government/academia	22	31.4	9	24.3	13	39.4
Industry	34	48.6	21	56.8	13	39.4
No funding	6	8.6	4	10.8	2	6.1
NA	8	11.4	3	8.1	5	15.2
Disease categories (Based on ICD-10 categories)						
I Certain infectious and parasitic diseases	4	5.7	2	5.4	2	6.1
II Neoplasms	18	25.7	7	18.9	11	33.3
IV Endocrine, nutritional and metabolic diseases	5	7.1	4	10.8	1	3.0
V Mental and behavioral disorders	3	4.3	0	0.0	3	9.1
IX Diseases of the circulatory system	17	24.3	8	21.6	9	27.3
X Diseases of the respiratory system	6	8.6	2	5.4	4	12.1
XIII Diseases of the musculoskeletal system and connective tissue	7	10.0	6	16.2	1	3.0
Others	1	1.4	1	2.7	0	0.0
NA	4	5.7	3	8.1	1	3.0
Intervention categories						
Pharmacological	38	54.3	25	67.6	13	39.4
Surgical	7	10.0	2	5.4	5	15.2
Treatment regimen	13	18.6	3	8.1	10	30.3
Management program	3	4.3	3	8.1	0	0.0
Prevention program	6	8.6	3	8.1	3	9.1
Screening	1	1.4	1	2.7	0	0.0
Devices	2	2.9	0	0.0	2	6.1

CEA: Cost-Effectiveness Analysis; CUA, Cost-Utility Analysis; NA, Not Available; ICD-10, International Classification of Diseases, 10th Revision.

### Methodologies of Included Studies

The majority of included studies (65.7%) reported patient baseline information. Nearly half of the studies (48.6%) did not report methods used to control for confounders, and matching (30.0%) was the most frequently used method for controlling, followed by regression (17.1%). Quality-adjusted life year (QALY) was the most frequently used effectiveness measure (55.7%), followed by the clinical endpoint (21.4%) and life year (18.6%). One-fifth of the studies included both direct and indirect costs (21.4%). The main sources of effectiveness were observational studies (48.6%), followed by registry and hospital information system (22.9%), while the main sources of cost were claims (31.4%), followed by the hospital information system (22.9%), and governmental published sources (18.6%). More than 70% of the studies did not report missing data of RWD. Among the studies with the report of missing data, excluding individuals with missing data was the most common method of handling the missing data, followed by imputation (25.0%). In addition, one study (5.0%) requested missing data from additional sources, while three studies reported missing data but did not use any method for handling. Half of the studies (52.9%) used a threshold to determine cost-effectiveness, and nearly one-third of the studies (17.1%) did not report any sensitivity analyses. The majority of the studies (82.9%) used a time horizon longer than 1 year, and nearly one-fifth of the studies (17.1%) did not report the time horizon. The number of discounted (52.9%) and undiscounted (47.1%) studies was about the same. (Table 2).

**TABLE 2:** The methodologies used of included studies.

Methodologies	Total*N* = 70		Analytic decision model*N* = 37		Non-analytic decision model*N* = 33	
	*N*	% (SD)	*N*	% (SD)	*N*	% (SD)
Patient baseline information						
Yes	46	65.7	19	51.4	27	81.8
No	24	34.3	18	48.6	6	18.2
Confounders controlled						
Randomization	3	4.3	1	2.7	2	6.1
Matching	21	30.0	8	21.6	13	39.4
Regression	12	17.1	1	2.7	11	33.3
NA	34	48.6	27	73.0	7	21.2
Analytic models						
Decision tree	4	10.8	4	10.8	-	-
Markov	30	81.1	30	81.1	-	-
Others	3	8.1	3	8.1	-	-
Effectiveness						
QALYs	39	55.7	30	81.1	9	27.3
DALYs	1	1.4	0	0.0	1	3.0
Life years	13	18.6	5	13.5	8	24.2
Clinical endpoint	15	21.4	2	5.4	13	39.4
Health care utilization	2	2.9	0	0.0	2	6.1
Cost input						
Only direct costs	55	78.6	27	73.0	28	84.8
Both direct and indirect costs	15	21.4	10	27.0	5	15.2
Sources of effectiveness						
Claims	16	22.9	10	27.0	6	18.2
Registry	9	12.9	2	5.4	7	21.2
Observational studies	11	15.7	2	5.4	9	27.3
Hospital information system	34	48.6	23	62.2	11	33.3
Sources of costs						
Claims	13	18.6	8	21.6	5	15.2
Registry	10	14.3	6	16.2	4	12.1
Literature review	22	31.4	9	24.3	13	39.4
Government-published resources	16	22.9	7	18.9	9	27.3
Observational studies	2	2.9	1	2.7	1	3.0
Hospital information system	7	10.0	6	16.2	1	3.0
Report of missing data						
Yes	20	28.6	6	16.2	14	42.4
No	50	71.4	31	83.8	19	57.6
Methods of handling missing data^a^						
Imputation	5	25.0	0	0.0	5	35.7
Excluding	11	55.0	3	50.0	8	57.1
Request from other sources	1	5.0	1	16.7	0	0.0
No	3	15.0	2	33.3	1	7.1
ICER Threshold						
Yes	37	52.9	27	73.0	10	30.3
No	33	47.1	10	27.0	23	69.7
Sensitivity analysis						
Only deterministic sensitivity analysis	15	21.4	7	18.9	8	24.2
Only probabilistic sensitivity analysis	20	28.6	12	32.4	8	24.2
Both deterministic and probabilistic sensitivity analysis	23	32.9	18	48.6	5	15.2
NA	12	17.1	0	0.0	12	36.4
Time horizon						
≤ 1 year	12	17.1	6	16.2	6	18.2
> 1 year	21	30.0	6	16.2	15	45.5
Lifetime	25	35.7	22	59.5	3	9.1
NA	12	17.1	3	8.1	9	27.3
Discount rate						
Yes	37	52.9	30	81.1	7	21.2
No	33	47.1	7	18.9	26	78.8
QHES score	92.4	7.0	95.7	5.4	88.7	6.8

NA, Not Available; QALY, Quality-Adjusted Life Year; DALY, Disability-Adjusted Life Year; ICER, Incremental Cost-Effectiveness Ratio; QHES, Quality of Health Economic Studies.

a The denominator is the 20 of studies with report of missing data.

### Comparison of Studies With or Without Decision-Analytic Models

**FIGURE 3:**
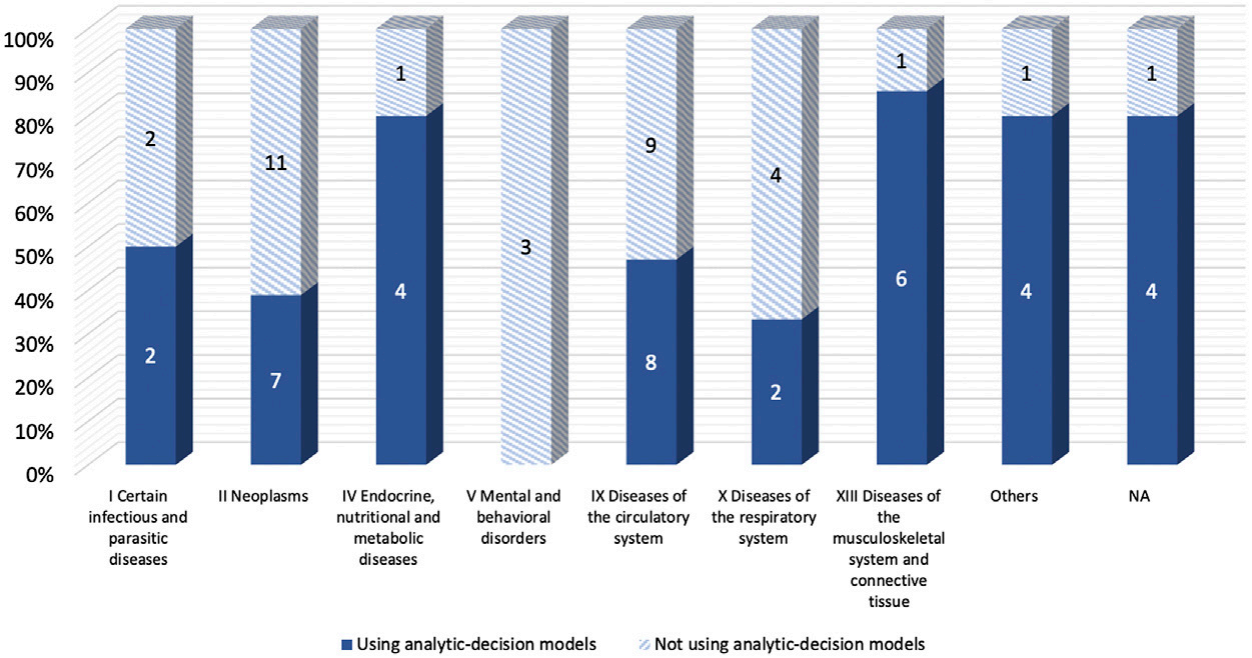
Differences in disease categories between real-world cost-effectiveness analysis with or without decision-analytic model.

**FIGURE 4:**
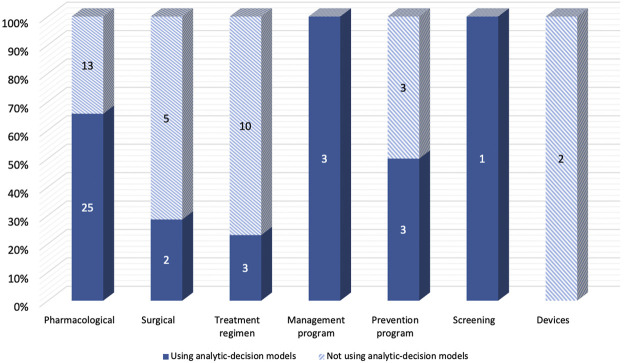
Differences in intervention categories between real-world cost-effectiveness analysis with or without decision-analytic model.

The majority of included studies with decision-analytic models used CUA (86.5%), while most of the studies without the model used CEA (78.8%). (Table 1). For the diseases evaluated in the studies, Figure 3 showed that Studies with decision analysis models were more likely to study on pharmacological interventions, management programs, and screening, while studies not based on decision analysis models were more likely to study on surgical interventions, treatment regimens, and devices. In terms of the interventions evaluated, Figure 4 illustrated that the studies with decision-analytic models preferred to evaluate pharmacological interventions, management programs, and screening, whereas those without models preferred to study surgical interventions, treatment regimens, and devices.

**FIGURE 5:**
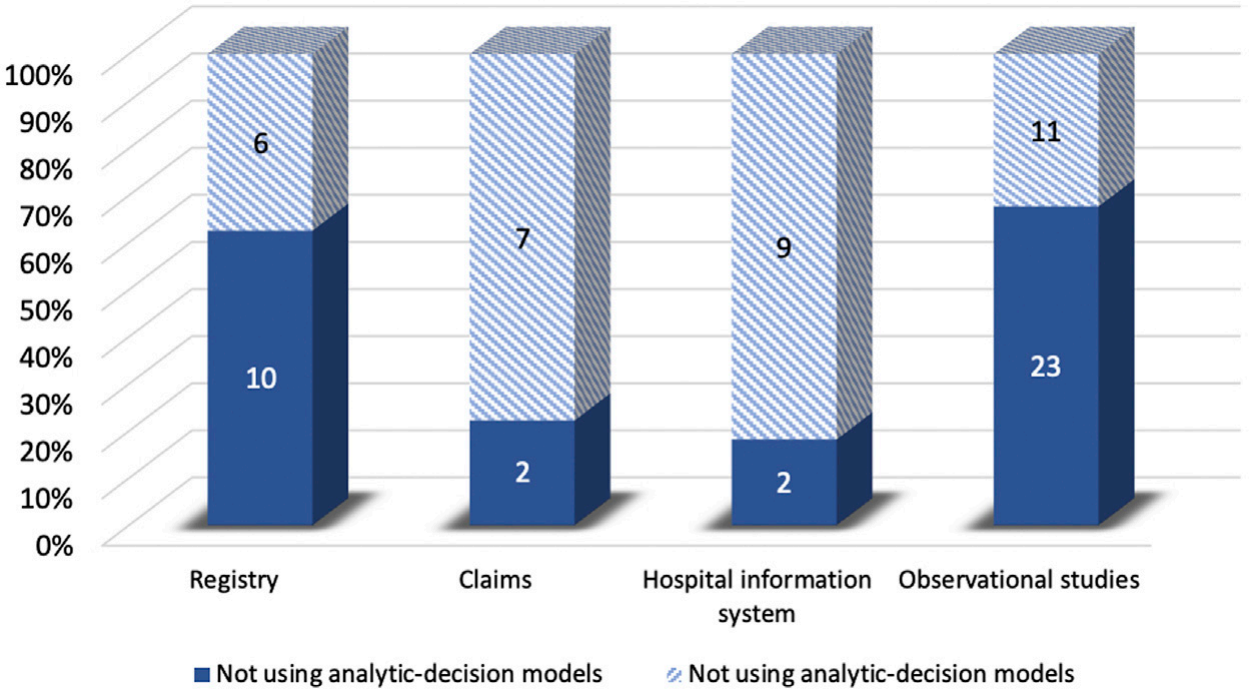
Differences in effectiveness sources between real-world cost-effectiveness analysis with or without decision-analytic model.

**FIGURE 6:**
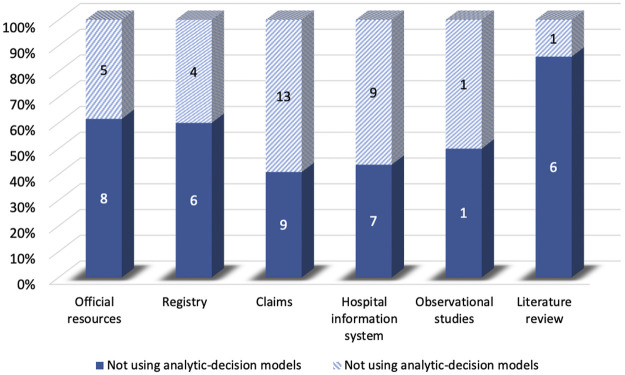
Differences in cost sources between real-world cost-effectiveness analysis with or without decision-analytic model.

Compared to the studies without decision-analytic models, those with the model were less likely to control for confounding variables and preferred to use QALYs as the effectiveness measure. However, the studies without the model were less likely to use threshold and sensitivity analysis, and the time horizon of them was shorter compared to the studies with the model. For sources of effectiveness, compared to the studies without decision-analytic models, the effectiveness of those with the model was less likely to be obtained from claims and health information systems and was more likely to be obtained from the registry and observational studies (Figure 5). As for sources of costs, Figure 6 demonstrated that official resources, registry, observational studies, and especially literature review were preferable for the studies using decision-analytic models, while claims and hospital information systems were preferable for those not using the models. In terms of missing data, the studies that did not use the model were more likely to report missing data. The methods of handling missing data were mainly excluding regardless of whether the model was used.

### Quality of Included Studies

**FIGURE 7:**
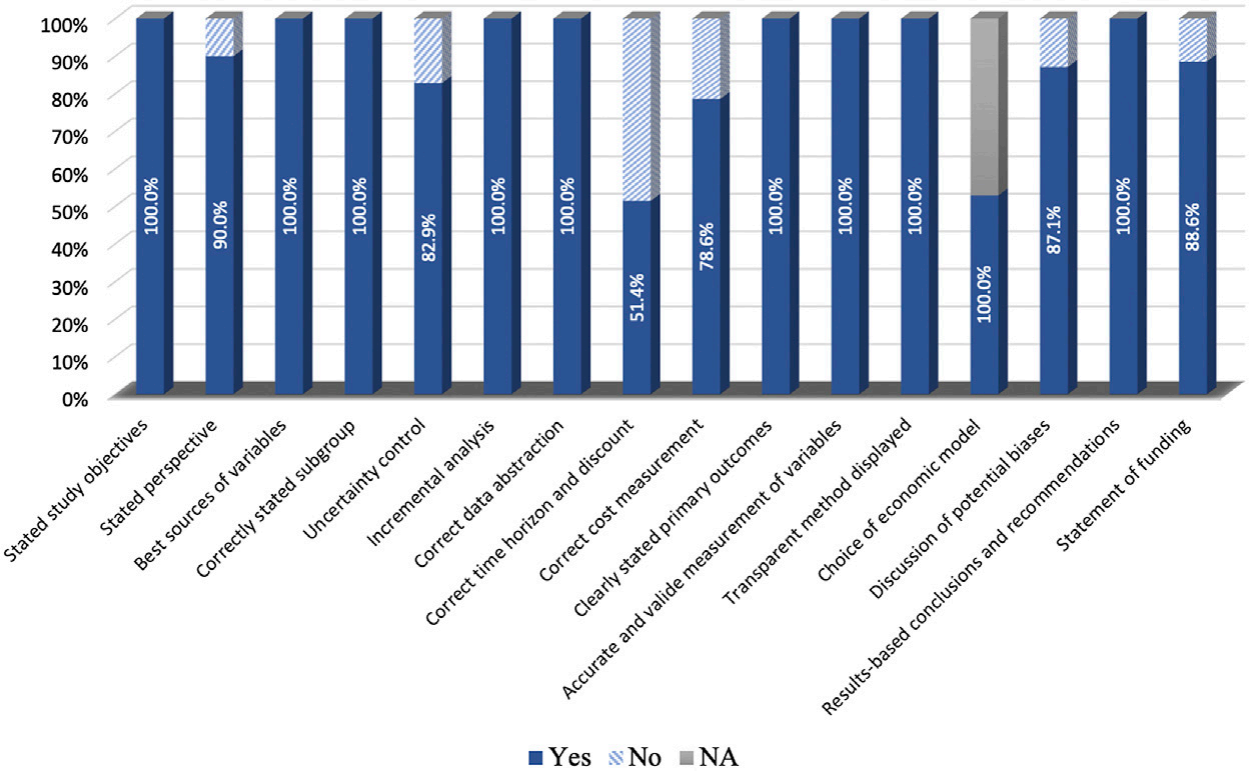
Quality assessment for the included studies.

The average QHES score for the studies with decision-analytic models was 95.7, while the score for the studies without the model was 88.7. The detailed results of the quality assessment are shown in Figure 7. Most of the included studies were conducted reasonably well. However, many studies have failed to deal with the time horizon, where only 51.4% of studies stated the time horizon and used discounting correctly.

## Discussion

This systematic literature review assessed the characteristics and methodologies of the CEA studies based on big data and RWD. Out of 70 included studies, we found that the number of the CEA studies based on big data and RWD has been increased over the years, and the majority of the studies were published between 2011 and 2020, which is similar to previous studies reviewing the economic evaluations based on RWD and routine data (Gansen, 2018; Parody-Rúa et al., 2020). We also found that the study region with the most stuides was Europe. This distribution is as expected given that many European countries have HTA agencies and have been using CEA studies to make reimbursement and formulary decisions (Dakin et al., 2015; Makady et al., 2018). Most of the first authors were from government or academic institutions, and most of the funding came from the industry, which is similar to a previous study (Parody-Rúa et al., 2020).

A study by Bowrin et al. systematically reviewed the barriers of using RWD in CEA modeling as well as the existing guidelines and recommendations for incorporating RWD in CEA modeling, in which they found that RWD is valuable in CEA studies for their internal information and suggested that the methods and potential applications of RWD in CEA should be studied (Bowrin et al., 2019). Our study complemented this research gap with a systematic review of the published CEA studies based on big data and RWD. Bowrin et al. indicated that there might be several barriers in the CEA studies using RWD, among which confounding bias was one of the main issues (Bowrin et al., 2019). Our findings are consistent with their results. In the 70 studies using RWD we included, we found that nearly half of the RWD-based studies lacked the control for confounders. Direct use of RWD may be biased due to possible differences in characteristics between the control and experimental groups and may result in causality not being explained. Confounders need to be tightly controlled in future studies using RWD. In future research, it is important to control for confounders and make the experimental group and the control group comparable. A similar issue is a lack of reporting baseline information of the study population. If there is a deviation between RWD and the baseline characteristics of the study population in the CEA, the direct use of the RWD data may be biased. The studies that did not report baseline information accounted for nearly of the studies that used the decision-analytic model. Although all of these studies used sensitivity analysis that could reduce the uncertainty, the reporting of the results of base case analysis might be biased. Bowrin et al. also mentioned that CEA using RWE might have the issue of missing data (Bowrin et al., 2019). During our review, we found that more than 70% of the included studies did not report missing data and how it was handled. This issue was more common in the CEA studies using the model. In future research, missing data should be strictly reported for CEA studies using RWD. Although Bowrin et al. indicated that the use of RWD might have a small sample size, in our review (Bowrin et al., 2019), we found that most the included studies had a sample size larger than 500, and even eight studies had a sample size of more than 10,000. However, there were more than a quarter of the included studies without reporting the sample size.

Compared with the previous study, we also compared the CEA study using RWD with and without the decision-analytic model. The CEA studies using models were more likely to study chronic diseases and to use a lifetime horizon, which might be due to the ability of decision-analytic models in simulating the lifetime cost-effectiveness (Drummond and McGuire, 2005; Tarride et al., 2010). Although CEA studies directly based on RWD can also provide long-term effectiveness and costs, they are rarely lifelong. However, in most of the studies using the model with a lifetime horizon, RWD-based effectiveness did not reach the lifetime. Although sensitivity analysis can partially solve this problem by reducing the uncertainty with a range of ICER, how to solve the potential problems of extrapolating the use of RWD still needs to be studied (Makady et al., 2018). In terms of the diseases studied, the studies without decision-analytic were more likely to study pharmacological interventions, while those with the model were more likely to focus on the treatment regimen. A direct comparison of pharmacological interventions without the model can reduce the uncertainty introduced by the model (Briggs, 2000). However, for the treatment regimen, it might be difficult to select a sample in a real-world setting where the treatment regimen is always complicated (Schulman et al., 2013). Consequently, those studies aiming to compare different treatment regimens preferred to use a model approach that allows greater freedom in the choice of study and control groups (Behar et al., 2017; Thronicke et al., 2020). This might be due to the fact that most of the studies with the model used sensitivity analysis to control for uncertainty. Compared to CEA studies with decision-analytic models, both the effectiveness and costs were more likely to be obtained from the literature review, which might be due to CEA studies using models often use mixed data from different sources (Briggs et al., 2006). In addition, the effectiveness of the studies using the decision-analytic model was mainly from claims and hospital information system, while the sources of studies without the model were more extensive. In addition to the above two, registry and observational studies were also main sources for studies without the model. We also found that four studies without models did not test the uncertainty of the study or control for confounders for assessing the effectiveness and costs (Olivares et al., 2008; Isla-Tejera et al., 2013; Tsai et al., 2018; Chan et al., 2020). As we discussed above, the lack of these methods could bring biases to results, and it is difficult to inform decision-making by deterministic results alone (Briggs, 2000; Parody-Rúa et al., 2020). Furthermore, in long-term CEA studies without the model, most of them were not discounted. Some of these studies even used a life-long time horizon (Liao et al., 2017; Wei et al., 2017). Without the discounting for the long-term of effectiveness and costs might overestimate the cumulative effectiveness and costs and might bias in ICER depends on a greater impact of the discounting on costs or effectiveness (Gravelle and Smith, 2001). In addition, we used the QHES to identify the quality of the included studies. Compared to the CHEERS, the QHES items have a better specificity, and QHES has a scoring system, which could facilitate the comparison of different studies (Ofman et al., 2003; Husereau et al., 2013). We found that the score of the studies included was higher than 75 regardless of whether the model was used or not, indicating a high quality of the studies.

In addition to the specific issues of using RWD, we also found some common problems related to CEA in the included research. The vast majority of CEA studies that adopted a social perspective included indirect costs. However, there were still several studies from a societal perspective that did not include indirect costs (Aarnio et al., 2015; van Leent et al., 2015; Dor et al., 2018; de Jong et al., 2019a; de Jong et al., 2019b; Voermans et al., 2019). Although none of these studies focusing on malignant diseases that can cause serious damage to the patient’s productivity or infectious diseases that can infect others (Aarnio et al., 2015; van Leent et al., 2015; Dor et al., 2018; de Jong et al., 2019a; de Jong et al., 2019b; Voermans et al., 2019), ignoring indirect costs to some extent underestimates the total costs and the benefits of productivity that could accrue to patients from more effective interventions (Drummond and McGuire, 2005). When using society as a research perspective of cost, opportunity cost instead of acquisition cost should be measured as the cost of interventions or programs. However, in all the studies that we included using society as the perspective, the costs were directly used acquisition costs, and there was no discussion why these costs were not adjusted into opportunity costs (Lindgren et al., 2009; Lekander et al., 2010; Yang et al., 2010; Lekander et al., 2013; Aarnio et al., 2015; Zhao et al., 2016; Tanaka et al., 2017; Vassall et al., 2017; Dor et al., 2018; de Jong et al., 2019a; de Jong et al., 2019b; Behan et al., 2019; Lin et al., 2019; Voermans et al., 2019; Wang et al., 2019; Arrobas et al., 2021). This might lead to the overestimation of costs and ICER. In addition, we found that most of the utilities used in the studies using decision-analytic models were derived from literature review, which might not suit the model population and result in potential biases. Although this limitation is widespread in research using decision-analytic models and not only limited to those studies using RWD, such a limitation should also be circumvented in order to improve the validity of the study.

However, in this study we found that less big data is used in CEA. When searching for literature, in order to avoid the inclusion of the studies only using RWD as one minor part of the data sources, we used a more rigorous search strategy. This might lead to a reduction in the scope of our included studies and might excluded some CEA studies that used big data. However, because many studies might have multiple data sources, especially the CEA studies using the decision-analytic model. Including all the studies where RWD were used might lead to too much literature and reduce the feasibility of the study. Given the potentials of big data, we encourage future CEA studies to use big data to support decision-making (Wordsworth et al., 2018). Big data are featured by high volume, high velocity, high variety, high value, and high veracity (Mehta and Pandit, 2018). Beyond the economic evaluation of diseases or interventions based on a cohort, big data can act as an important role in personalized precision health economics and outcomes research (p-HEOR) (Chen et al., 2020). Advanced predictive algorithms of applying big data such as natural language processing (NLP) and machine learning (ML) should be used more in CEA studies and other economic evaluations (Fahr et al., 2019). Given the potentials of big data, we encourage future CEA studies to use big data to support decision-making.

According to the trend in the publications, the number of CEA studies using RWD is likely to continue increasing over the next decades. The 21st Century Cures Act passed in 2016 emphasized the use of RWD to support regulatory decision making, including the approval of new indications for approved drugs, and a series of guidance was launched later (Hudson and Collins, 2017). It is not difficult to imagine that over the next decades, more and more CEA studies will use RWD. In the case of big data, over the next decades, relevant CEA research using big data is likely to emerge, but not on a large scale, given that few mature algorithms and related methods are available (Fahr et al., 2019). Big data have far-reaching potential for prediction and could be used in some long-term CEA studies to replace some of the current methods to predict long-term effectiveness and costs (Fahr et al., 2019; Chen et al., 2020).

Although there is no systematic guideline on the use of RWE in CEA, there is some guidelines about using RWD from certain sources. Deidda et al. published a framework on the use of natural experiments, and some of the items contained therein are similar to the problems we found, which might be helpful to guide future CEA research using natural experiments as RWD sources to avoid methodology problems (Deidda et al., 2019). However, considering that there are more and more researches using RWD, there is still a need for systematic guidance on using RWD.

Some study limitations are worth mentioning. First, although the searching strategies used various terms, only a restricted set of synonyms was utilized within the systematic search. However, the terms used in the searching strategies are comparable to other reviews regarding the cost-effectiveness analysis. Second, since many diseases, as well as interventions, were included in the study, we did not compare the result of CEA studies based on big data and RWD to that of CEA studies based on RCT, because there were too much CEA literature using RCT data or mixed data for each disease or intervention, and it was difficult to ensure that all the literature can be fully included, for which the results of comparing might be biased. In future research, it is needed to conduct systematic reviews comparing the result of CEA studies based on big data and RWD to that of CEA studies based on RCT for a specific disease or intervention, in which the published literature can be covered completely. Third, the searching terms were restricted in the title, abstract, and keywords. Some studies based on big data and RWD but not mentioned in each respective field might have been missed. In addition, although our research found that there were eight studies with a sample size of more than 10,000, according to our definition of big data, these were not classified as studies using big data. When doing this research, there was no specific definition of big data in the HTA. Therefore, during the search process, we used definitions that if two or more RWD were combined in a single parameter, or if any artificial intelligent methods were used to process the data, which might limit the economic evaluation that we can include on the use of big data to a certain extent. Future research specifically on big data is still needed to enrich the review of this type of research. Fourth, this study only focused on CEA studies, and did include CBA studies, because we were concerned that if CBA was included, there might be some differences from CEA when extracting methodology or results. Future studies are needed for CBA studies using RWD. Finally, only full-text studies in English were included in the review, resulting in the disqualification of published studies that met other inclusion criteria.

## Conclusion

A total of 70 studies were identified in this systematic literature review regarding cost-effectiveness analysis based on big data and real-world data. The review shows that big data and RWD have been increasingly applied in conducting the cost-effectiveness analysis. However, few CEA studies are based on big data characterized by 5Vs. The characteristics and methodologies were described and compared between the studies with decision-analytic models as well as the ones without the model. In future CEA studies using big data and RWD, it is encouraged to control confounders and to discount in long-term research when decision-analytic models are not used.

## Data Availability

The original contributions presented in the study are included in the article/Supplementary Material, further inquiries can be directed to the corresponding authors.
