# Corrigendum: Big Data and Real-World Data Based Cost-Effectiveness Studies and Decision-Making Models: A Systematic Review and Analysis

**DOI:** 10.3389/fphar.2021.833827

**Published:** 2022-01-25

**Authors:** Z. Kevin Lu, Xiaomo Xiong, Taiying Lee, Jun Wu, Jing Yuan, Bin Jiang

**Affiliations:** ^1^ Department of Clinical Pharmacy and Outcomes Sciences, University of South Carolina, Columbia, SC, United States; ^2^ Department of Pharmaceutical and Administrative Sciences, Presbyterian College School of Pharmacy, Clinton, SC, United States; ^3^ Department of Clinical Pharmacy, School of Pharmacy, Fudan University, Shanghai, China; ^4^ Department of Administrative and Clinical Pharmacy, School of Pharmaceutical Sciences, Health Science Center, Peking University, Beijing, China

**Keywords:** big data, real-world data, cost-effectiveness analysis, pharmacoeconomics, systematic review

In the original article, there was an error in the **3rd paragraph** in the **Discussion** section. In this paragraph of the original text, we included the sentence “Furthermore, in long-term CEA studies without the model, most of them were not discounted. Some of these studies even used a life-long time horizon (Armeni et al., 2016; Liao et al., 2017; Wei et al., 2017).” Here, the context may mislead readers that Armeni et al., 2016’s study did not use an analytic model and discount, but in fact, while they used a life-time horizon, their study used an analytic model and discounted the long-term outcomes in the model. Therefore, the citation for Armeni et al., 2016 was removed and the corrected sentence is “Furthermore, in long-term CEA studies without the model, most of them were not discounted. Some of these studies even used a life-long time horizon (Liao et al., 2017; Wei et al., 2017).”

The authors apologize for this error and state that this does not change the scientific conclusions of the article in any way. The original article has been updated.

