# Supplementary material for: Big Data and Real-World Data based Cost-Effectiveness Studies and Decision-making Models: A Systematic Review and Analysis
Source: Front Pharmacol. 2021 Oct 19;12:700012. doi: 10.3389/fphar.2021.700012 (PMC8562301; doi:10.3389/fphar.2021.700012)
Supplement: Supplementary file 1 [file DataSheet1.docx]

## Supplementary Material S1: Search strategies

**Database: Medline (via PubMed), searched June 3, 2020**

| **Search** | **Search terms** | **Results** |
| --- | --- | --- |
| #1 | ((('cost-utility analysis'[Title/Abstract]) OR ('cost-effectiveness analysis'[Title/Abstract])) OR ('economic evaluation'[Title/Abstract])) OR (Pharmacoeconomics[Title/Abstract]) | 19,781 |
| #2 | ((((((('big data'[Title/Abstract]) OR ('real-world'[Title/Abstract])) OR ('real-world study'[Title/Abstract])) OR ('real-world evidence'[Title/Abstract])) OR ('real-world data'[Title/Abstract])) OR (RWD[Title/Abstract])) OR (RWE[Title/Abstract])) OR (RWS[Title/Abstract]) | 44,999 |
| #3 | ((('electronic health records'[Title/Abstract]) OR ('EHRs'[Title/Abstract])) OR (claims[Title/Abstract])) OR (registry[Title/Abstract]) | 150,069 |
| #4 | #2 OR #3 | 190,831 |
| #5 | #1 AND #4 | 1,066 |

**Database: Embase, searched June 3, 2020**

| **Search** | **Search terms** | **Results** |
| --- | --- | --- |
| #1 | 'cost-utility analysis':ti,ab OR 'cost-effectiveness analysis':ti,ab OR 'economic evaluation':ti,ab OR Pharmacoeconomics:ti,ab | 31,317 |
| #2 | 'big data':ti,ab OR 'real-world':ti,ab OR 'real-world study':ti,ab OR 'real-world evidence':ti,ab OR 'real-world data':ti,ab OR RWD:ti,ab OR RWE:ti,ab OR RWS:ti,ab | 76,921 |
| #3 | 'electronic health records':ti,ab OR 'ehrs':ti,ab OR claims:ti,ab OR registry:ti,ab | 266,543 |
| #4 | #2 OR #3 | 331,987 |
| #5 | #1 AND #4 | 1,746 |

**Database: Web of Science, searched June 3, 2020**

| **Search** | **Search terms** | **Results** |
| --- | --- | --- |
| #1 | TI = (cost-effectiveness analysis* or cost-utility analysis* or economic evaluation* or Pharmacoeconomics*) | 22,161 |
| #2 | AB = (cost-effectiveness analysis* or cost-utility analysis* or economic evaluation* or Pharmacoeconomics*) | 61,531 |
| #3 | TI = (big data* or real-world* or real-world study* or real-world evidence* or real-world data* or RWD or RWE or RWS) | 44,583 |
| #4 | AB = (big data* or real-world* or real-world study* or real-world evidence* or real-world data* or RWD or RWE or RWS) | 222,466 |
| #5 | TI = (electronic health records* or EHRs or claims or registry) | 70,186 |
| #6 | AB = (electronic health records* or EHRs or claims or registry) | 308,479 |
| #7 | #1 or #2 | 75,413 |
| #8 | #3 or #4 | 246,969 |
| #9 | #5 or #6 | 354,103 |
| #10 | #8 OR #9 | 594,155 |
| #11 | #7 AND #10 | 2,781 |

**Database: Cochrane Library, searched June 3, 2020**

| **Search** | **Search terms** | **Results** |
| --- | --- | --- |
| #1 | ('cost-utility analysis' OR 'cost-effectiveness analysis' OR 'economic evaluation' OR Pharmacoeconomics):ti,ab | 21,212 |
| #2 | ('big data' OR 'real-world' OR 'real-world study' OR 'real-world evidence' OR 'real-world data' OR RWD OR RWE OR RWS):ti,ab | 5,527 |
| #3 | ('electronic health records' OR 'EHRs' OR claims OR registry):ti,ab | 17,064 |
| #4 | #2 or #3 | 20,526 |
| #5 | #1 and #4 | 1,158 |

## Supplementary Material S2: Quality of Health Economic Studies Instrument

| Items | Questions |
| --- | --- |
| 1 | Was the study objective presented in a clear, specific, and measurable manner? |
| 2 | Were the perspective of the analysis (societal, third-party payer, etc.) and reasons for its selection stated? |
| 3 | Were variable estimates used in the analysis from the best available source (i.e., randomized control trial - best, expert opinion - worst)? |
| 4 | If estimates came from a subgroup analysis, were the groups prespecified at the beginning of the study? |
| 5 | Was uncertainty handled by (1) statistical analysis to address random events, (2) sensitivity analysis to cover a range of assumptions? |
| 6 | Was incremental analysis performed between alternatives for resources and costs? |
| 7 | Was the methodology for data abstraction (including the value of health states and other benefits) stated? |
| 8 | Did the analytic horizon allow time for all relevant and important outcomes? Were benefits and costs that went beyond 1 year discounted (3% to 5%) and justification given for the discount rate? |
| 9 | Was the measurement of costs appropriate and the methodology for the estimation of quantities and unit costs clearly described? |
| 10 | Were the primary outcome measure(s) for the economic evaluation clearly stated and did they include the major short-term was justification given for the measures/scales used? |
| 11 | Were the health outcomes measures/scales valid and reliable? If previously tested valid and reliable measures were not available, was justification given for the measures/scales used? |
| 12 | Were the economic model (including structure), study methods and analysis, and the components of the numerator and denominator displayed in a clear, transparent manner? |
| 13 | Were the choice of economic model, main assumptions, and limitations of the study stated and justified? |
| 14 | Did the author(s) explicitly discuss direction and magnitude of potential biases? |
| 15 | Were the conclusions/recommendations of the study justified and based on the study results? |
| 16 | Was there a statement disclosing the source of funding for the study? |

**Reference:** Ofman JJ, Sullivan SD, Neumann PJ, Chiou CF, Henning JM, Wade SW, et al. Examining the value and quality of health economic analyses: implications of utilizing the QHES. J Manag Care Pharm. (2003) 1:53-61. doi: 10.18553/jmcp.2003.9.1.53.
